# Leptin signals via TGFB1 to promote metastatic potential and stemness in breast cancer

**DOI:** 10.1371/journal.pone.0178454

**Published:** 2017-05-25

**Authors:** Ameet K. Mishra, Christopher R. Parish, Ma-Li Wong, Julio Licinio, Anneke C. Blackburn

**Affiliations:** 1 ACRF Department of Cancer Biology and Therapeutics, The John Curtin School of Medical Research, The Australian National University, Canberra, ACT Australia; 2 Mind and Brain Theme, South Australian Health and Medical Research Institute, Adelaide, SA, Australia; 3 School of Medicine, Flinders University, Bedford Park, Adelaide, SA, Australia; University of South Alabama Mitchell Cancer Institute, UNITED STATES

## Abstract

Epidemiological studies have shown obesity to be linked with poorer outcomes in breast cancer patients. The molecular mechanisms responsible for the increased risk of invasive/metastatic disease with obesity are complex, but may include elevated levels of adipokines such as leptin. Using physiological levels of leptin found in obesity in a novel chronic in vitro treatment model (≤200 ng/ml for 14 days), we confirmed the occurrence of leptin-mediated changes in growth, apoptosis and metastatic behavior, and gene expression changes representing epithelial-to-mesenchymal transition (EMT) and a cancer stem cell (CSC) like phenotype in breast epithelial and cancer cell lines (MCF10A, MCF10AT1, MCF7 and MDA-MB-231). Further, we have discovered that these effects were accompanied by increased expression of TGFB1, and could be significantly reduced by co-treatment with neutralizing antibody against TGFB1, indicating that the induction of these characteristics was mediated via TGFB1. Occurring in both MCF7 and MCF10AT1 cells, it suggests these actions of leptin to be independent of estrogen receptor status. By linking leptin signalling to the established TGFB1 pathway of metastasis / EMT, this study gives a direct mechanism by which leptin can contribute to the poorer outcomes of obese cancer patients. Inhibitors of TGFB1 are in currently in phase III clinical trials in other malignancies, thus identifying the connection between leptin and TGFB1 will open new therapeutic opportunities for improving outcomes for obese breast cancer patients.

## Introduction

Breast cancer is the second-leading cause of cancer-related deaths among women worldwide [1]. Epidemiological studies have shown that obese and overweight women have poorer outcomes in breast cancer [2, 3]. In fact, obesity is linked with many aspects of breast cancer including higher prevalence, higher grade tumors, higher rates of metastatic, recurrent, or drug resistant disease, and higher relative risk of death [4–7]. Both breast cancer and obesity are multi-factorial diseases and no single factor is responsible for the promotion of tumor progression in obese patients [8].

At the cellular level, tumor cells exist in a complex environment composed of many diverse cells, and tumor invasion results in immediate proximity of breast cancer cells to adipocytes in the mammary gland [9–11]. Cancer associated adipocytes are the source of a number of secreted factors including leptin, IL6, IL1β and estrogen, and these factors have been independently linked with breast cancer progression [12]. Leptin is a pleiotropic hormone primarily synthesized by adipose tissues and circulating levels are higher in obese people (>100 ng/ml) than non-obese people (5–50 ng/ml) [13, 14]. Some population studies have linked high levels of leptin to increased risk of breast cancer, independent of obesity indices [15, 16], and a meta-analysis of 23 studies of leptin levels and breast cancer concluded there is an association between higher circulating levels of leptin with breast cancer occurrence and progression [17]. The contribution of leptin to breast cancer development is evident from pre-clinical studies where mice deficient in leptin or with dysfunctional leptin receptors did not develop transgene-induced mammary tumors [18, 19] and had reduced growth of transplanted tumors[20]. Numerous studies have previously shown that leptin can directly act on breast cancer cells and modulate their behavior, including promoting proliferation, transformation and reducing apoptosis (reviewed in [21]), however the effect of leptin on the metastatic process is relatively poorly understood.

The epithelial-to-mesenchymal transition (EMT) is often activated during metastasis and is directly linked to the acquisition of cancer stem cell (CSC) properties [22]. CSCs can mediate metastasis and are associated with drug resistance and poorer clinical outcomes [23, 24]. There are numerous signalling pathways involved in EMT including MAPK/PI3K, TGFB1/SMAD and Wnt/β-catenin [25]. Leptin activates a number of signalling pathways that have been previously described as important in cancer promoting events and that overlap with the induction of EMT, including MAPK and Wnt/β-catenin [26–28], however leptin signalling through the TGFB1 pathway, a major player in EMT, has not been demonstrated in breast cancer.

We have examined the effect of chronic, physiological leptin treatment on the metastatic and CSC-like characteristics of breast epithelial and cancer cells, and have discovered that promotion of invasiveness and CSC behavior by leptin is mediated through binding of TGFB1 to its receptor, increasing the likely contribution of leptin signalling to poorer outcomes in obesity, and adding to the list of pathways that may be readily targeted in obese cancer patients.

## Materials and methods

### Cell culture

Breast epithelial and cancer cells were obtained from American Type Culture Collection (ATCC, Manassas, USA) at the start of this project, and experiments were completed within 35 passages of the cell growth. MCF7 and MDA-MB-231 cells were maintained in DMEM containing phenol red, 10% fetal bovine serum (FBS), 1% combination of penicillin, streptomycin and neomycin (PSN). MCF10A and MCF10AT1 cells were cultured in DMEM/F12 containing phenol red, 5% (MCF10A) or 2.5% (MCF10AT1) horse serum, 0.01mg/ml insulin, 500ng/ml hydrocortisone, 20ng/ml epidermal growth factor and 1% PSN. During treatments, serum content of the media was decreased to 2% (low serum media).

### Cell proliferation and apoptosis

For proliferation assays, cells were treated for 72hr (acute treatment) with leptin or vehicle in low serum media and the rate of cell proliferation was estimated with Vybrant MTT cell proliferation assay kit (Invitrogen). Cell numbers were estimated from a standard curve. For apoptosis, cells were treated for 24hr with leptin or vehicle in low serum media, trypsinized and collected with media to avoid loss of apoptotic/dead floating cells. Apoptotic cells were measured with Annexin V-FITC/PI kit (Invitrogen). Data was acquired on LSR Fortessa Cell Analyzer (BD Biosciences) and analyzed with Flowjo software (Treestar Flowjo, Version 10.0.6) and presented as % apoptotic cells. Experiments were performed in triplicate, and at least 3 independent experiments conducted.

### Chronic leptin treatment conditions

Obesity is a chronic disorder, thus to mimic this, long term treatment of cells was used in these studies, unless otherwise stated. Conditions were based on two publications, Mani et al. and Morel et al., which studied the induction of EMT in mammary epithelial cells [22, 29]. Low serum media was used during chronic treatment to decrease the factors present in serum which may have a background effect or that can form complexes with recombinant proteins used in this study. Breast cancer cells were seeded in normal culture media and cultured overnight. Medium was then replaced with low (2%) serum media. Cells were again cultured overnight before addition of treatments (vehicle, leptin (200 ng/ml), TGFB1 (2.5 ng/ml) and neutralizing antibody against TGFB1 (5 ng/ml): Ab-TGFB1 (Sigma-Aldrich)). Low serum media with treatment was changed every third day and cells were sub-cultured once they reached 70–80% confluence. Treatment was continued for 14 days, and cells were harvested and replated as appropriate for each assay.

### Hanging drop assay

The hanging drop assay measures the ability of cells to aggregate, an important property of non-invasive epithelial cells [30]. Cells were trypsinized and resuspended in DMEM containing 10% FBS at 1x10^6^ cells/ml. Cells (20μl) were placed on the lid of a 6-well plate. The lid was inverted and placed on the plate that contained media to maintain humidity and prevent evaporation. Cells were cultured overnight and then photographed using Olympus 1X71 at 40x objective (three different fields of view per drop). At least three drops per treatment were performed, and experiments repeated at least 3 times. The area of individual aggregates formed (size range approximately 50–200μm) was measured using NIH ImageJ software and expressed as pixels^2^.

### Matrigel invasion assay

Cells were trypsinized and resuspended in DMEM containing 1% FBS at 1x10^5^ cells/ml. Cells (5,000) were seeded into cell inserts (Sigma-Aldrich) pre-coated with matrigel (BD Biosciences). The lower chamber was filled with DMEM containing 10% FBS. After 24hr, cells were fixed, stained with Giemsa stain and cells that had invaded the matrigel were counted. Treatments were performed in triplicate, and experiments repeated at least 3 times. Data is expressed as a number of invaded cells/5,000 cells.

### Cell migration

After 14 days of treatment, cells were trypsinized, resuspended and seeded at equal densities in 96-well plates in standard medium and allowed to adhere overnight. Treatments (eg. leptin, 200 ng/ml in low serum) were then continued until cells reached 100% confluence (usually overnight). Confluent cells were scratched with a 96-pin wound maker (Essen Biosciences, Michigan, USA), plates were washed twice with media to remove any cell debris, and wells replenished with low serum media containing treatments. Cells were scanned inside an incubator every 2 hr for 24 hr with Incucyte Kinetic Imaging System (IncuCyte 2011A, Essen Biosciences). The rate of cell migration (μm/hr) was calculated over the first 12 hr, well within the population doubling time for these cells to minimize the impact of any proliferative effects of treatments on the assay.

### Mammosphere formation assay

After 14 days of leptin treatment (200 ng/ml), cells were trypsinized, resuspended and seeded at 50,000 cells per well in 6-well ultra-low attachment plates (Costar). Cells were grown in specialized medium containing mammocult proliferation supplement (StemCell Technologies, British Columbia, Canada), heparin (StemCell Technologies, British Columbia, Canada) and 1% methylcellulose (Sigma-Aldrich, MO, USA). Leptin treatment was continued during mammosphere formation. Mammospheres were counted after 7 days and data was expressed as number of colonies per 5,000 cells seeded. Experiments were performed in triplicate and repeated independently at least 3 times.

### Quantitative polymerase chain reaction

Total RNA was extracted with Qiazol and RNeasy Mini kit (Qiagen, Maryland, USA). DNA was removed using DNase (Qiagen). RNA was converted to cDNA using Omniscript Reverse Transcriptase kit and Oligo(dT)_12-16_ kit (Qiagen). Predesigned primers sequences were obtained from PrimerBank (Table 1). qPCR was performed with SYBR green Mastermix (Applied Biosystems) on 7900HT Fast Real-Time PCR system (Applied Biosystems) using the amplification protocol: 50°C for 2 minutes, 95°C for 10 minutes, followed by 40 cycles at 95°C for 15 seconds and 60°C for 1 minute. Dissociation curve analysis was also applied to confirm specific amplification. Expression levels were normalized to the house-keeping gene hypoxanthine-guanine phosphoribosyltransferase (*HPRT1*) and results were analyzed as relative expression to *HPRT1*. Where results involve leptin treatment, they are presented as fold-change compared to untreated cells. Each experiment included at least 3 biological replicates, and results shown are representative of at least 3 independent experiments with consistent outcomes. In some cases, biological replicates from independent experiments were combined for statistical analysis.

**Table 1:** Sequences of primers used for quantitative RT-PCR.

Name	NCBI gene symbol	Primer sequence
Hypoxanthine phosphoribosyltransferase 1	HPRT1	F. 5’-CCTGGCGTCGTGATTAGTGAT-3’
		R. 5’-AGACGTTCAGTCCTGTCCATAA-3’
Glyceraldhyde-3-phosphate dehydrogenase	GAPDH	F. 5’-CATGAGAAGTATGACAACAGCCT-3’
		R. 5’-AGTCCTTCCACGATACCAAAGT-3’
Leptin receptor (functional, long form)	LEPR	F. 5’-TGCCACCAAATTCAACCTATGAC-3’
		R. 5’-TGCTCACTCCGAAAGCAACAG-3’
Adiponectin receptor 1	ADIPOR1	F. 5’-TCCTGCCAGTAACAGGGAAG-3’
		R. 5’-AGGGGAAGTGTCAGTACCCG-3’
Adiponectin receptor 2	ADIPOR2	F. 5’-CCCTCTCTTACAAGCCCATCA-3’
		R. 5’-CCGACCTTCCCATACCTTACAAA-3’
Cadherin 1, type 1, epithelial cadherin	CDH1	F. 5’-CGAGAGCTACACGTTCACGG-3’
		R. 5’-GGGTGTCGAGGGAAAAATAGG-3’
Snail homolog 2 (SLUG)	SNAI2	F. 5’-ATACCACAACCAGAGATCCTCA-3’
		R. 5’-GACTCACTCGCCCCAAAGATG-3’
Signal transducer and activator of transcription 3	STAT3	F.5’-CAGCAGCTTGACACACGGTA-3’
		R.5’-AAACACCAAAGTGGCATGTGA-3’
Jun proto-oncogene	C-JUN	F.5’-GTCCTTCTTCTCTTGCGTGG-3’
		R.5’-GGAGACAAGTGGCAGAGTCC-3’
FBJ murine osteosarcoma viral oncogene homolog	C-FOS	F.5’-GTGGGAATGAAGTTGGCACT-3’
		R.5’-CTACCACTCACCCGCAGACT-3’
Transcription growth factor, β receptor 1	TGFBRI	F. 5’-GCTGTATTGCAGACTTAGGACTG-3’
		R. 5’-TTTTTGTTCCCACTCTGTGGTT-3’
Transcription growth factor, β receptor 2	TGFBRII	F. 5’-GACATCAATCTGAAGCATGAGAACA-3’
		R. 5’-GGCGGTGATCAGCCAGTATT-3’
SMAD family member 2	SMAD2	F. 5’-GCTGTTTTCCTAGCGTGGCTT-3’
		R. 5’-TCCAGACCCACCAGCTGACT-3’
SMAD family member 3	SMAD3	F. 5’-CGGCAGTAGATGACATGAGG-3’
		R. 5’-TCAACACCAAGTGCATCACC -3’

### Western blotting

Western blotting was performed by standard protocols with enhanced chemiluminescence (ECL) detection. Protein was extracted from cells using MPER extraction buffer (Thermo Scientific, #78503). Protease inhibitors (Roche, #04 693 159 001) were used in all samples while phosphatase inhibitor (Calbiochem, #524629) was used when checking phosphorylated proteins (P-STAT3). Total protein was estimated using BCA protein assay kit (Thermoscientific, #23225). Equal amounts of protein (50μg) were separated by SDS-PAGE and transferred to PVDF membrane. The membrane was blocked with 5% BSA and specific proteins detected with the following primary antibodies: anti-leptin receptor (Abcam, #104403), anti-STAT3 (Cell Signalling, #9132), anti-phospho-STAT3 (Tyr705) (Cell Signalling, #9131) and anti-β-actin (loading control, Abcam, #Ab8227). Anti-rabbit IgG, HRP-linked secondary antibody (Cell Signalling, #7074) was used for ECL detection.

### FACS analyses

Fluorescence activated cell sorting (FACS) readings were acquired on LSR Fortessa cell analyser (BD Biosciences) and analysed with Flowjo software (Treestar Flowjo, Version 10.0.6). Experiments were performed in triplicate and repeated independently at least 3 times.

#### Aldefluor assay

Aldehyde dehydrogenase (ALDH) is the enzyme required for conversion of retinol to retinoic acid and is highly expressed in cells with stem like properties[23]. ALDH activity was assessed using the Aldefluor assay kit (StemCell Technologies, British Columbia, Canada). After 14 days of treatment, cells were resuspended at 10^6^ cells/ml in aldefluor buffer. Aldefluor reagent (5ul) was added and cells were incubated in the dark at 37°C for 45min. Cells were washed, resuspended in 500ul of aldefluor buffer, and intracellular fluorescence measured by FACS and presented as % aldefluor positive cells. A negative control of diethylaminobenzaldehyde (DEAB) was set in parallel to control for background fluorescence.

#### CD44/CD24 CSC markers

The CD44+/CD24- subset of human breast cancer cells are considered a CSC population of cells [31]. Anti-CD44-APC (BD Biosciences, #559942, 20μl/10^6^ cells) and anti-CD24-PE (BD Biosciences, #555428, 10–20μl/10^6^ cells) were used to estimate the percentage of CD44+/CD24- cells in cultured populations. After 14 days of treatment, cells were resuspended in FACS buffer (PBS, 1% FBS) with antibodies for 40 min on ice on shaker in the dark. After washing, fluorescence readings were acquired and data was expressed as % CD44+/CD24- cells.

#### TGFB1 expression

TGFB1 expression was measured with FACS. After 14 days of treatment, cells were fixed with neutral buffered formalin and permeabilized with 1% SDS. PE-Mouse anti-human TGFB1 (BD Biosciences, #562339, 5μl/10^6^ cells) was added and incubated in the dark at room temperature for 40min. After washing and resuspending in FACS buffer, fluorescence readings were acquired and data was expressed as % high TGFB1 expressing cells.

### Statistical analysis

GraphPad software (GraphPad Prism, version 6) was used to perform statistical analysis. Data are presented as mean ± SEM with the sample size, type of statistical analysis and P values presented in the figure legends.

## Results

### Breast cancer cells express LEPR and increase proliferation in response to leptin

**Fig 1:**
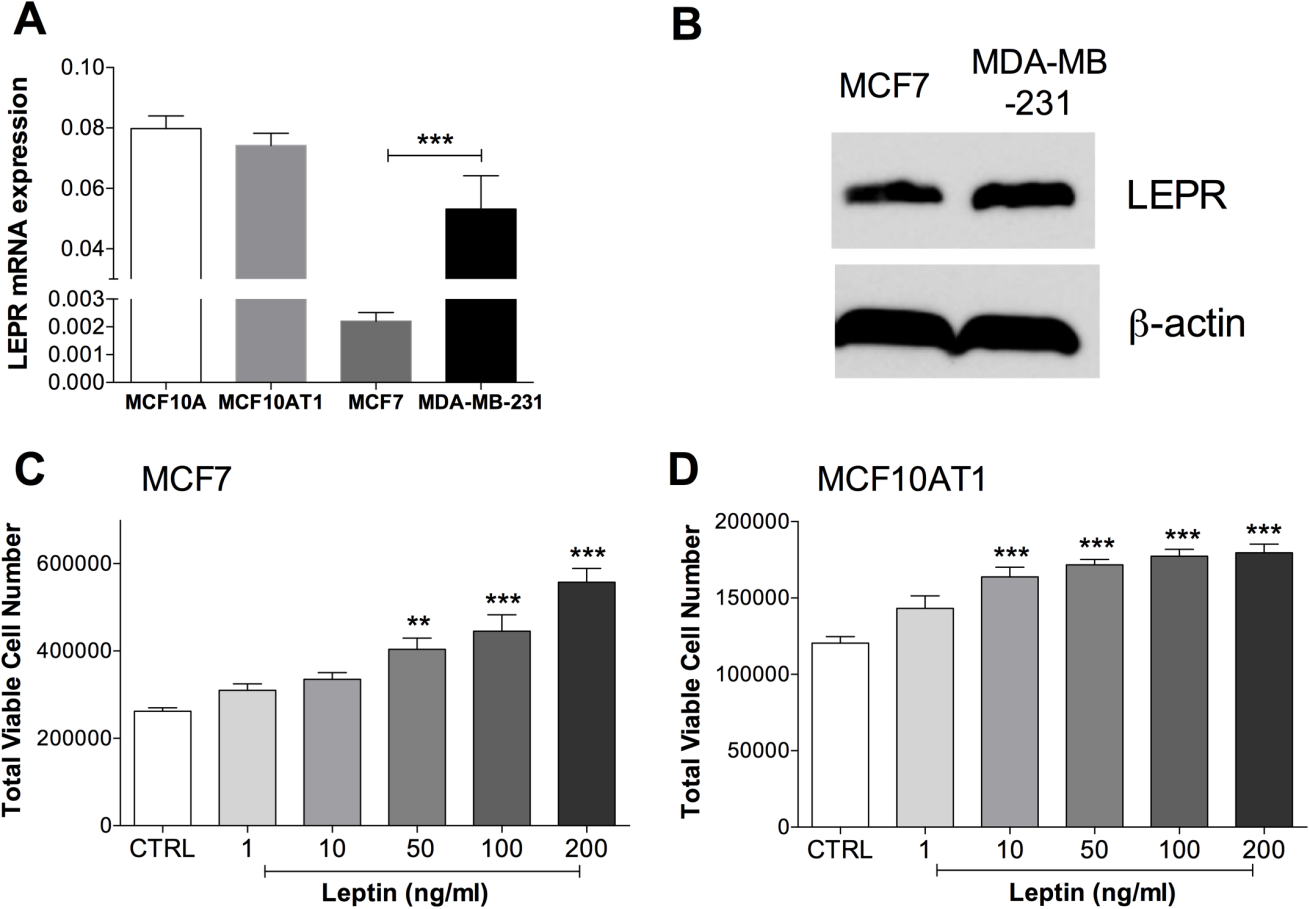
Breast cancer cells express LEPR and increase proliferation in response to leptin. **(a)** *LEPR* mRNA (qPCR, relative to *HPRT1*) and **(b)** LEPR protein expression (western blot) in breast epithelial cell lines. (Student’s t-test was used to compare differences in mRNA levels between pairs of cell lines—MCF10A vs MCF10AT1 and MCF7 vs MDA-MB-231. n = 9–11 wells combined from several experiments.) ***P<0.001. **(c)** MCF7 and **(d)** MCF10AT1 cells increased proliferation in response to 72 hr of acute leptin treatment, as measured by MTT assay. (n = 6–8 wells. ANOVA was used to compare among groups with Tukey’s multiple comparison test. **P<0.01, ***P<0.001 compared to control (CTRL)).

We first examined expression of the adipokine receptors, LEPR (functional leptin receptor), ADIPOR1 and ADIPOR2 (adiponectin receptors) in four cell lines chosen to represent different states of progression or subtypes of breast cancer: MCF10A (immortal, non-cancerous), MCF10AT1 (isogenic cancerous pair to MCF10A, transformed in vitro), MCF7 (luminal, hormone receptor positive, non-metastatic) and MDA-MB-231 (basal, hormone receptor negative, metastatic). *LEPR* mRNA was expressed in all cell lines (Fig 1A). MCF10A and MCF10AT1 cells did not differ in *LEPR* mRNA expression, whereas higher expression of *LEPR* mRNA (Fig 1A) was found in MDA-MB-231 compared to MCF7 cells. To determine if mRNA differences were reflected in protein levels, LEPR protein was investigated by western blotting in the two patient-derived breast cancer cell lines where mRNA levels differed the most. The mRNA difference was reflected in protein expression, although to a much lesser extent, and while MCF7 cells showed the lowest mRNA levels, LEPR protein was readily detected by western blotting (Fig 1B). *ADIPOR1* and *ADIPOR2* mRNA were detectable in all cell lines, but there were no remarkable differences in expression (data not shown).

As our hypothesis was that leptin would increase metastatic cell behaviour, we focused on the less invasive MCF7 and MCF10AT1 cells. To confirm that the breast cancer cells were responsive to leptin in our culture conditions, we measured cell proliferation as leptin has been shown to induce proliferation in a variety of cells [32–34]. A dose-dependent increase in cell proliferation after 3 days of acute leptin treatment was observed in both MCF7 and MCF10AT1 cells, with 200 ng/ml leptin resulting in 2.12-fold (MCF7) and 1.5-fold (MCF10AT1) more cells than controls (Fig 1C and 1D). Leptin treatment made a small but not significant decrease in the background percentage of apoptotic cells in both MCF7 and MCF10AT1 cells, such that the increase in cell number could not be accounted for by decreased background apoptosis in this time frame (data not shown).

### Chronic leptin decreased cell aggregation, and increased cell migration and invasion

**Fig 2:**
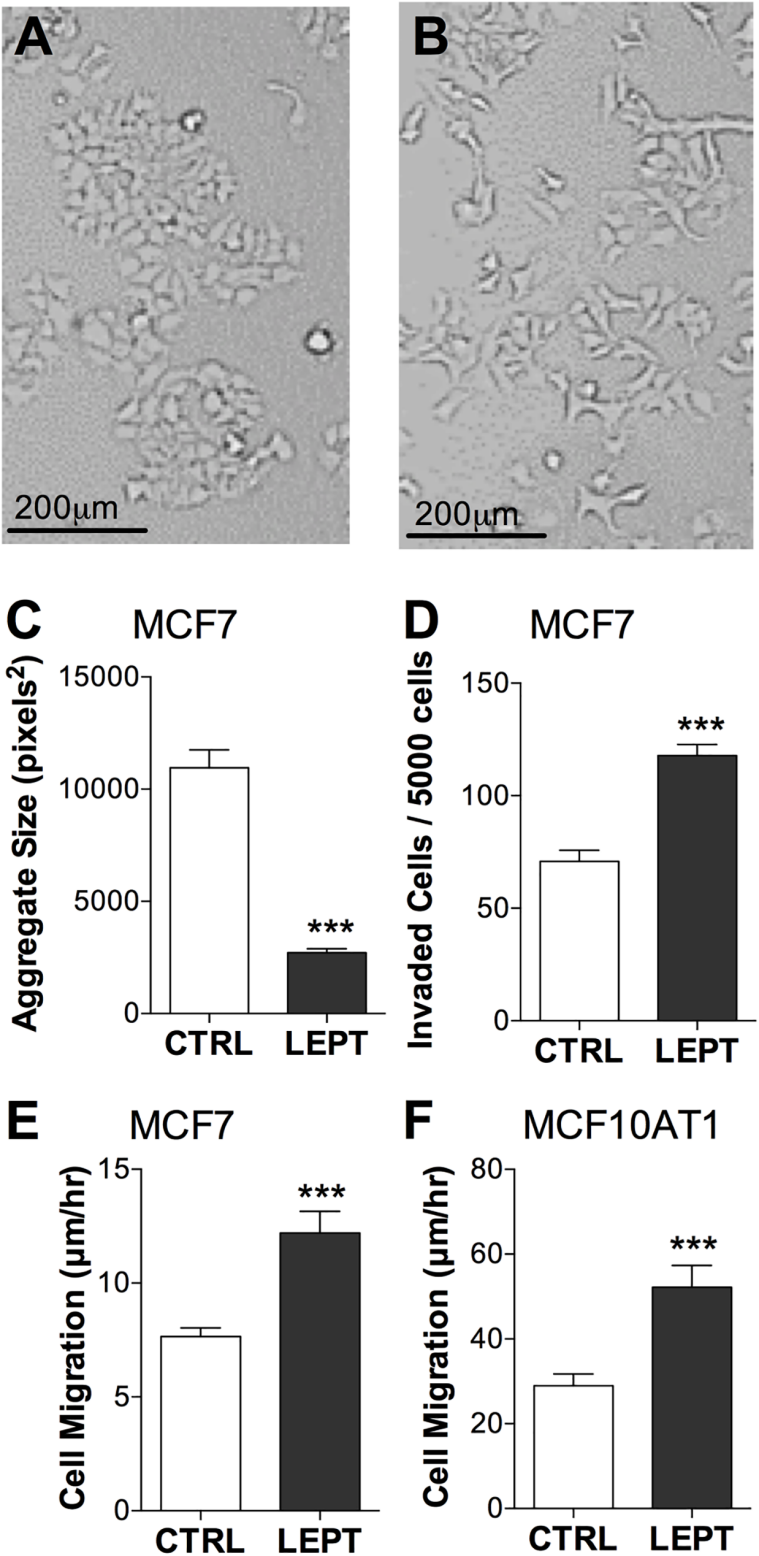
Leptin decreased cell aggregation, and increased cell migration and invasion. MCF7 or MCF10AT1 cells in low serum media were treated for 14 days with 200 ng/ml leptin (LEPT) and compared to untreated controls (CTRL). (**a**) CTRL and (**b**) LEPT-treated MCF7 cells are morphologically different. **(c)** Effect on MCF7 cells by hanging drop cell aggregation assay. ***P<0.001 (n = 5 drops, Student’s t-test. Data shown is from one representative experiment of 3 independent experiments.) **(d)** Effect on invasion of MCF7 cells into matrigel. ***P<0.001 (n = 18–20 wells, Student’s t-test, data combined from 3 independent experiments). **(e) and (f)** Effect on migration rate of **(e)** MCF7 and **(f)** MCF10AT1 cells measured by wound healing scratch assay. ***P<0.001 (n = 17–24 wells, Student’s t-test, data combined from several independent experiments).

Obesity is a chronic disorder, thus we examined the morphology of breast cancer cells after 14 days of 200 ng/ml leptin treatment in low serum conditions for changes consistent with increased invasiveness and EMT. After leptin treatment, MCF7 cells were more dispersed and more spindle-like in structure, with less cell-to-cell attachment than control cells (Fig 2A and 2B). Similar changes were also observed in MCF10AT1 cells (images not shown). The acquisition of a mesenchymal phenotype was assessed with functional assays. Leptin significantly decreased the size of MCF7 cell aggregates (Fig 2C), almost doubled the number of MCF7 cells invaded into matrigel (Fig 2D) and significantly increased the migration speed of MCF7 and MCF10AT1 cells (Fig 2E and 2F).

### Leptin decreased epithelial and increased mesenchymal and CSC characteristics

**Fig 3:**
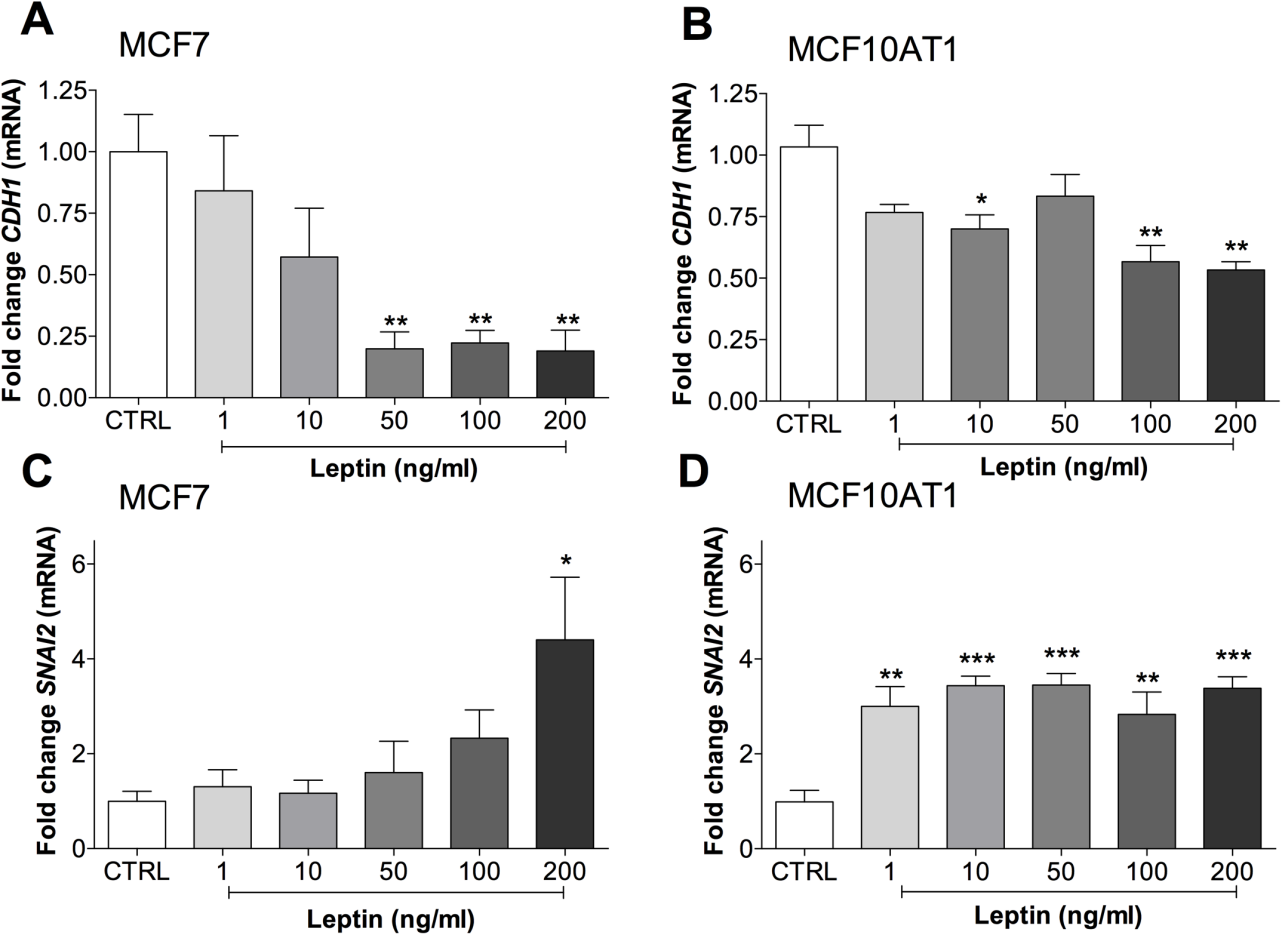
Leptin induced epithelial to mesenchymal transition indicated by mRNA markers. Effect of leptin treatment on mRNA expression of *CDH1* (**a and b**, n = 8 and 3) and *SNAI2* (**c and d**, n = 5 and 9) in MCF7 **(a and c)** or MCF10AT1 **(b and d)** cells. qPCR was normalised to *HPRT1* expression and is presented as fold-change compared to untreated control (CTRL). ANOVA was used to compare among groups with Tukey’s multiple comparison test. *P<0.05, **P<0.01, ***P<0.001 compared to CTRL.

To examine the loss of epithelial characteristics at the molecular level, changes in the mRNA expression of *CDH1* (E-cadherin) and *SNAI2* (snail family zinc finger 2) in MCF7 and MCF10AT1 cells after chronic leptin treatment were measured by qPCR. Loss of epithelial characteristics were indicated by significantly decreased *CDH1* mRNA expression (Fig 3A and 3B) while gain of mesenchymal characteristics were indicated by an increase in *SNAI2* (Fig 3C and 3D). These changes were dose-dependent in MCF7 cells.

**Fig 4:**
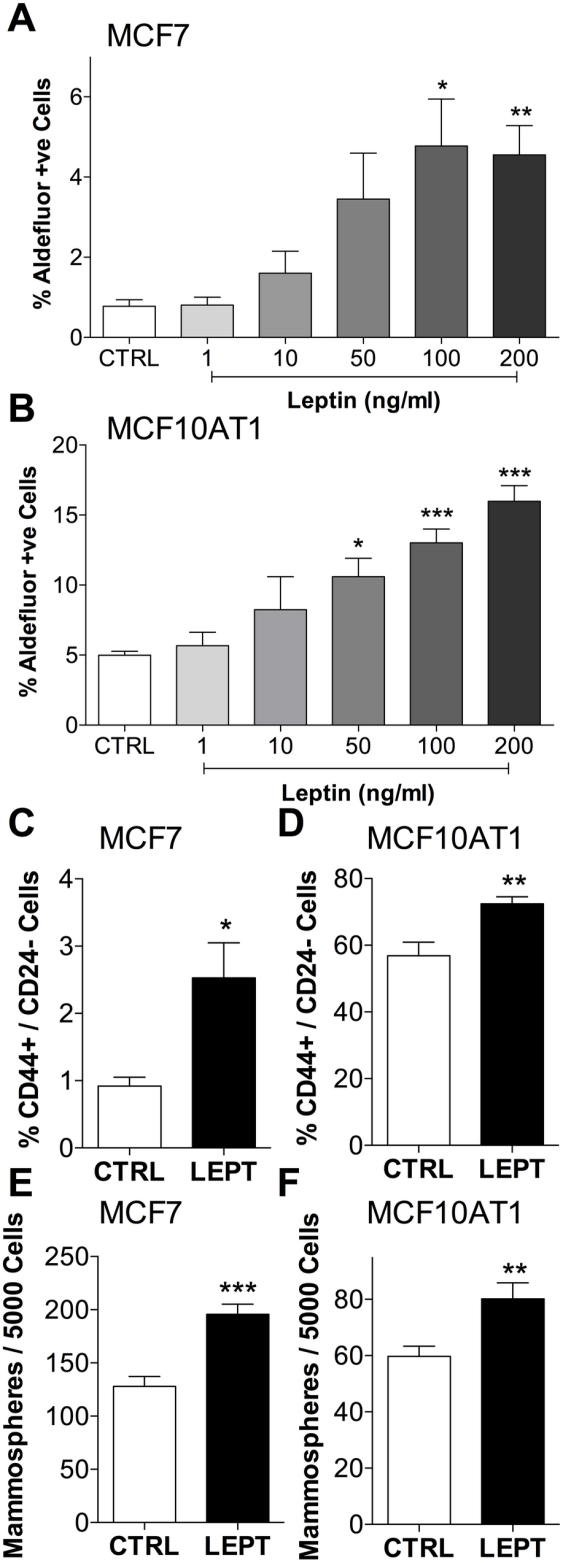
Leptin increased stem cell characteristics of breast cancer cells. Effect of leptin treatment (14 d in low serum conditions) on: aldefluor activity in **(a)** MCF7 (n = 7) and **(b)** MCF10AT1 (n = 6) breast cancer cells; CD44+/CD24- cell population in **(c)** MCF7 (n = 6) and **(d)** MCF10AT1 (n = 6) cultures; mammosphere formation by **(e)** MCF7 (n = 6) and **(f)** MCF10AT1 (n = 8) cells. (c-f) Leptin treatment was 200 ng/ml. (ANOVA was used to compare among groups with Tukey’s multiple comparison test (a and b). Student’s t-test was used to compare two groups (c-f).) *P<0.05, **P<0.01, ***P<0.001 compared to CTRL.

An EMT can be considered intermediate in the process of stem cell development [22]. We examined whether leptin-induced changes included changes to breast CSC markers. Leptin treatment significantly increased the percentage of aldefluor positive cells in both MCF7 and MCF10AT1 cells in a dose dependent manner (Fig 4A and 4B). Similarly, the CSC population as defined by the cell surface markers CD44+/CD24- was significantly increased in both breast cancer cell lines by leptin (Fig 4C and 4D). As the expression of these CSC markers is highly variable across cells lines, we functionally tested the stemness of the breast cancer cells using the mammosphere formation assay. Leptin treated MCF7 and MCF10AT1 breast cancer cells formed significantly more mammospheres than control cells (Fig 4E and 4F). These results indicate that chronic leptin treatment of breast cancer cells can directly alter their characteristics in a manner consistent with the more aggressive phenotype and poorer outcomes that are observed in obese patients.

### TGFB1 expression was induced by leptin and mediated leptin-induced changes

**Fig 5:**
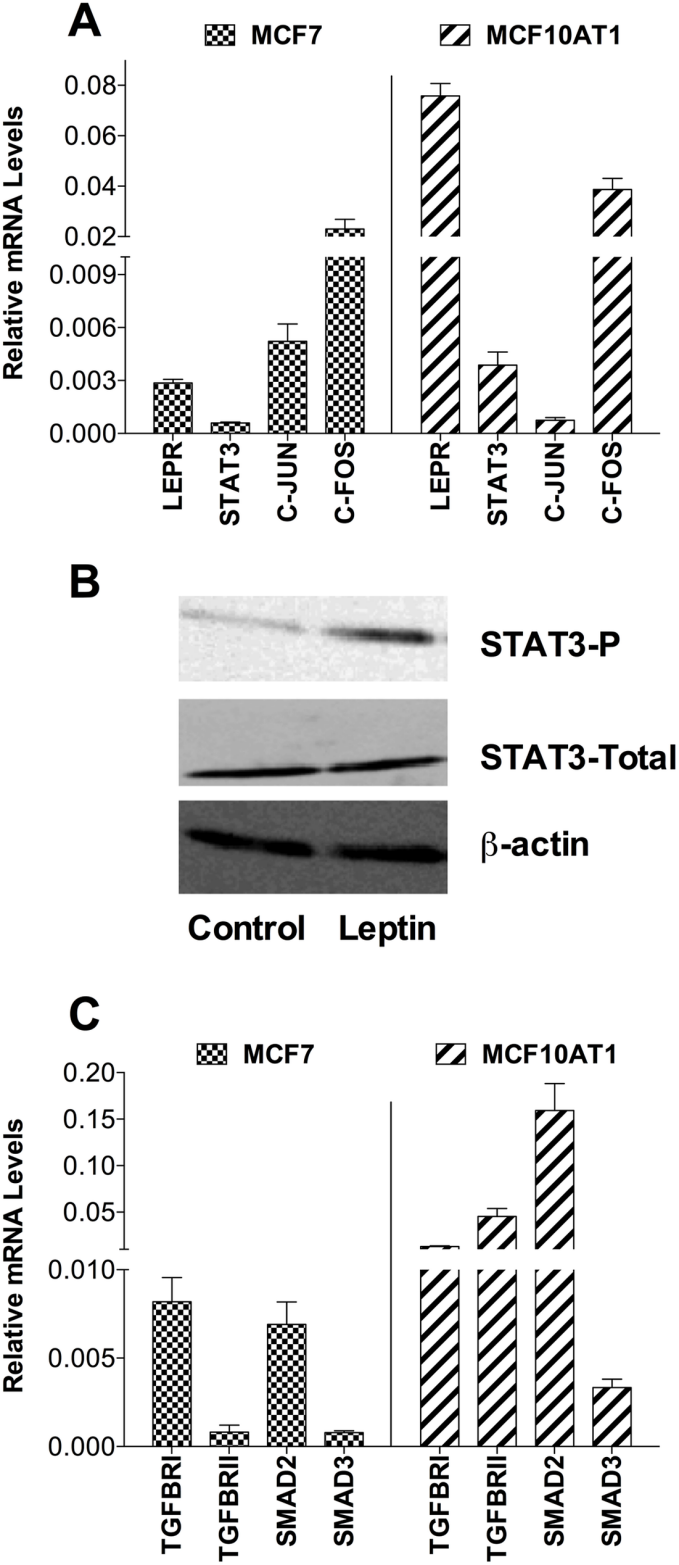
Leptin and TGFB signalling components are present. **(a)** mRNA expression of components of LEPR signalling: *LEPR*, *STAT3*, *C-JUN*, and *C-FOS*; **(b)** Effect of leptin treatment (200 ng/ml, 14d, low serum) on phosphorylated STAT3 (STAT3-P) levels (with total STAT3 and β-actin loading controls) in MCF7 cells by western blotting. **(c)** mRNA expression of components of TGFB1 signalling: *TGFBR1*, *TGFBRII*, *SMAD2*, *SMAD3*; in MCF7 and MCF10AT1 cells (n = 6 per group) (qPCR, relative to *HPRT1*).

**Fig 6:**
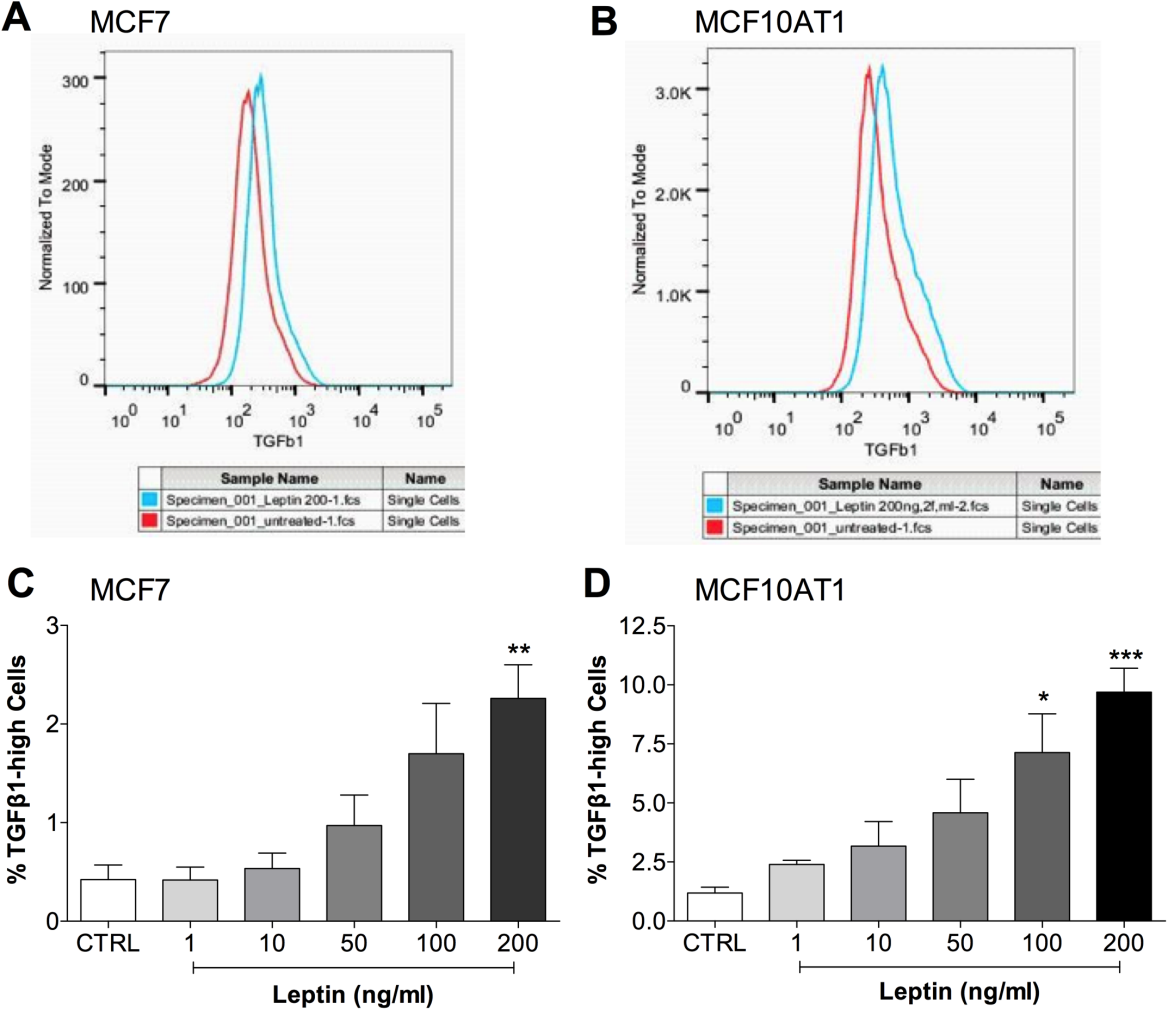
Increased TGFB1 protein expression in response to leptin. **(a and b)** FACS histograms showing increased intracellular TGFB1 levels in **(a)** MCF7 and **(b)** MCF10AT1 cells after chronic 200 ng/ml leptin treatment. (Red line–untreated control, Blue line–Leptin treated 200 ng/ml for 14 days.) **(c and d)** Dose responses for TGFB1 protein levels after leptin treatment in **(c)** MCF7 (n = 6) and **(d)** MCF10AT1 (n = 6) cells. (ANOVA was used to compare among groups with Tukey’s multiple comparison test.) *P<0.05, **P<0.01, ***P<0.001 compared to CTRL.

TGFB1 is a central player in EMT and cross-talks with other EMT signalling pathways. Thus, we examined the role of TGFB1 in leptin-induced EMT and CSC changes. In Fig 1A and 1B, we demonstrated the presence of LEPR on breast cancer cells. To investigate the presence of functional LEPR and TGFB1 signalling, the downstream components of these signalling pathways were measured at the mRNA level by qPCR. *LEPR*, *STAT3*, *JUN* (c-Jun), *FOS* (c-Fos) were expressed in both MCF7 and MCF10AT1 breast cancer cell lines (Fig 5A) indicating the presence of LEPR signalling components in these cells, while western blotting after chronic leptin treatment indicated increased STAT-3 phosphorylation, demonstrating active leptin signalling (Fig 5B), consistent with the observed proliferation effect (Fig 1). Similarly, components of TGFB1 signalling, *TGFBR1*, *TGFBR2*, *SMAD2*, *SMAD3* were expressed in both MCF7 and MCF10AT1 cells indicating the possible presence of intact TGFB1 signalling (Fig 5C). The effect of leptin treatment on intracellular TGFB1 protein levels was estimated by FACS. Leptin treatment resulted in an overall increase in TGFB1 protein levels (Fig 6A and 6B) and the percentage of cells with high anti-TGFB1 fluorescence was significantly increased in a dose-dependent manner, with 5.4-fold and 7.1-fold increases observed in MCF7 and MCF10AT1 cells respectively after 200 ng/ml leptin treatment (Fig 6C and 6D).

**Fig 7:**
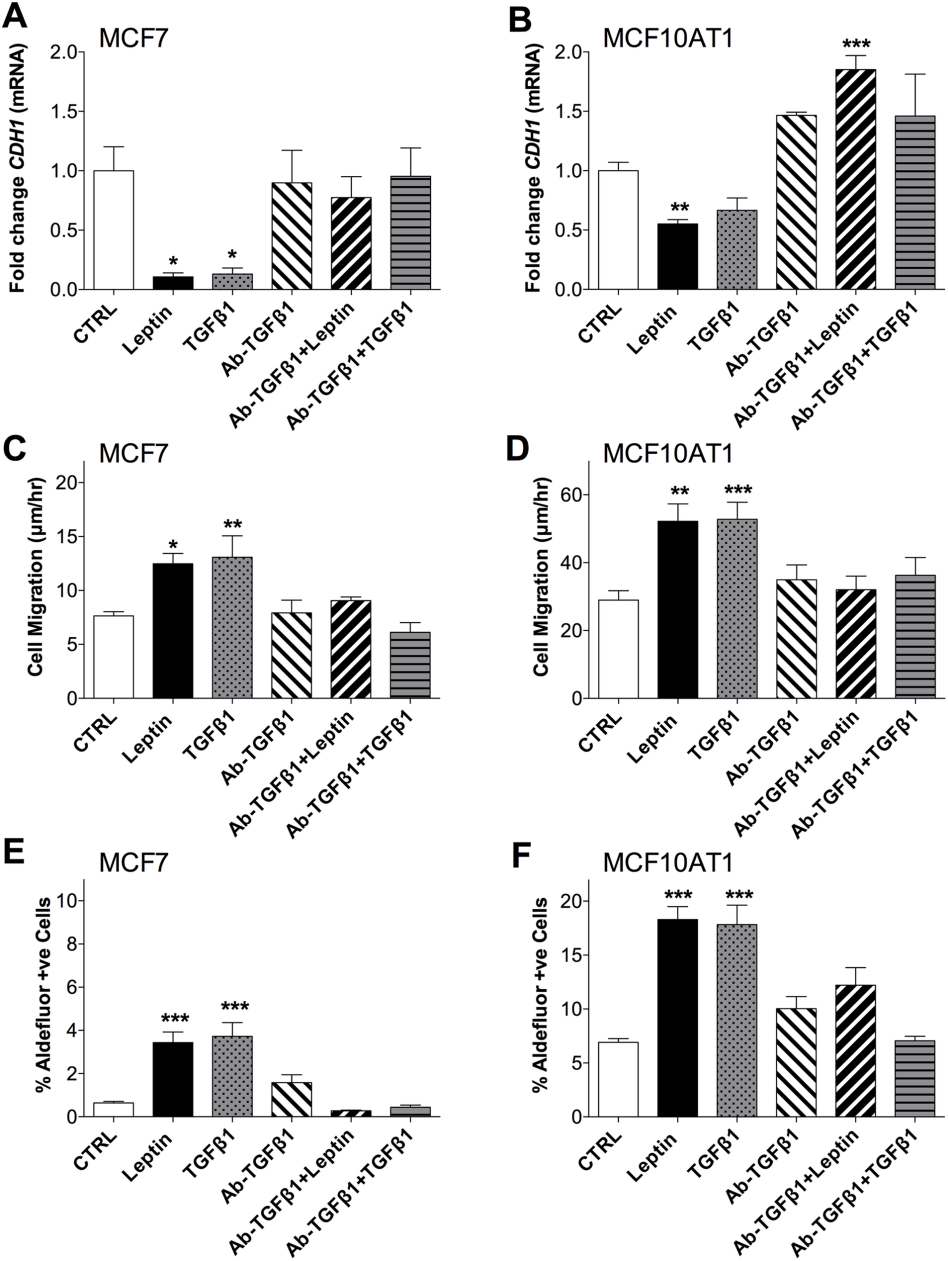
Neutralizing antibody against TGFB1 blocks leptin mediated actions. Effect of leptin (200 ng/ml), TGFB1 (2.5 ng/ml), Ab-TGFB1 (5 ng/ml), Ab-TGFB1+Leptin or Ab-TGFB1+TGFB1 treatment (all treatments for 14 days) compared with untreated controls on: **(a and b)** *CDH1* mRNA expression in **(a)** MCF7 & **(b)** MCF10AT1 cells (qPCR was normalised to *HPRT1* expression and is presented as fold-change compared to untreated control (CTRL)); **(c and d)** cell migration rate of **(c)** MCF7 and **(d)** MCF10AT1 cells; and **(e and f)** aldefluor activity of **(e)** MCF7 and **(f)** MCF10AT1 cells. (ANOVA was used to compare among groups with Tukey’s multiple comparison test. Data was combined from 2–3 experiments representative of at least 3 independent experiments.) *P<0.05, **P<0.01, ***P<0.001 compared to CTRL.

**Fig 8:**
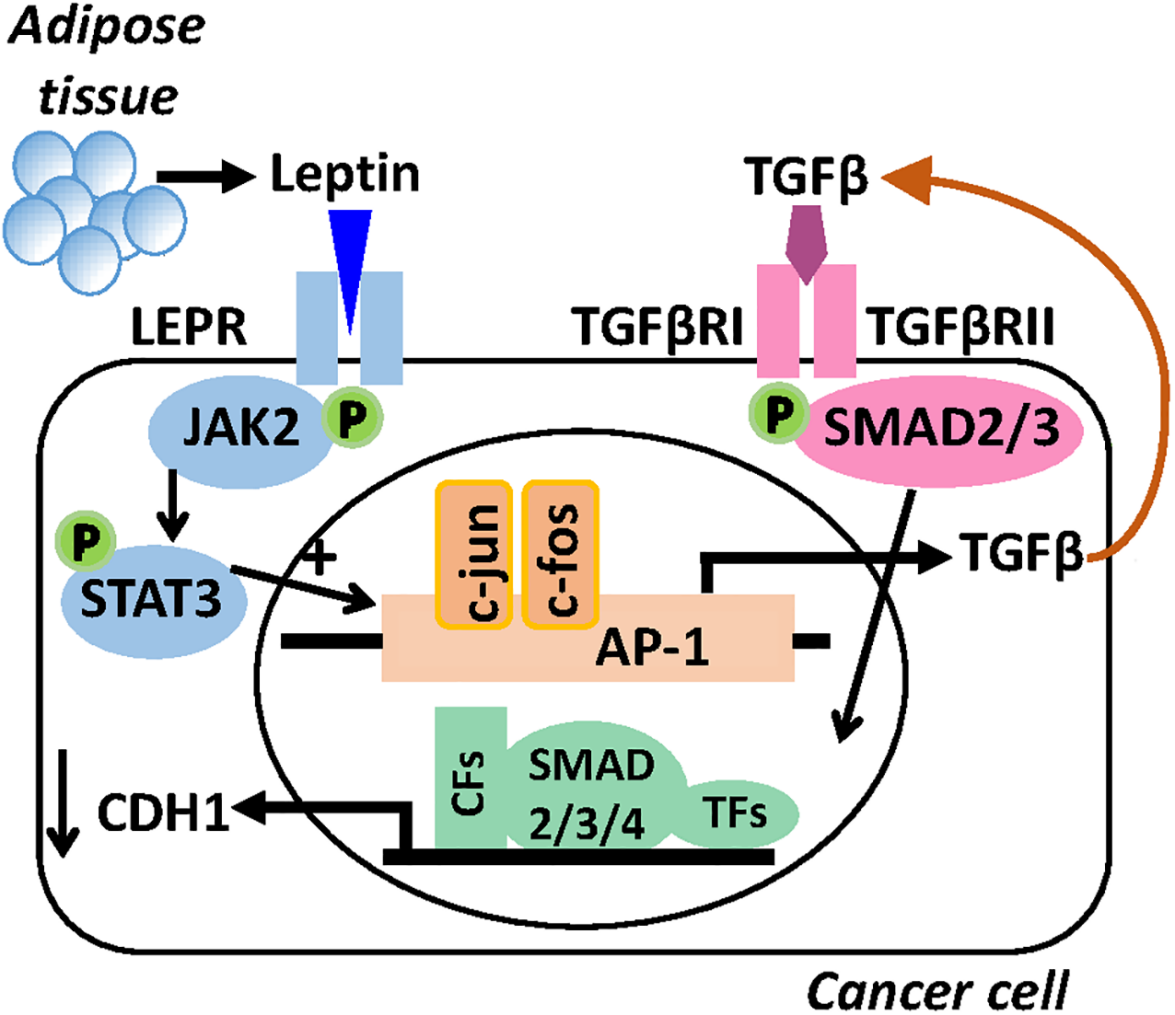
Schematic summary of the proposed autocrine / paracrine TGFB1 signalling in breast cancer. Leptin synthesized and secreted by adipose tissues (or cancer associated fibroblasts) will bind to the leptin receptor (LEPR) on cancer cells, activating JAK/STAT3 signalling. c-Jun, c-fos and AP-1 complex are recruited to the promoter of the *TGFB1* gene, increasing synthesis of TGFB. TGFB1 is secreted and binds to its receptors (TGFBRI and TGFBRII) expressed on cancer cells and activates SMAD2/3, recruiting SMAD2/3/4 to the nucleus with Transcription Factors (TFs) and co-factors (CFs), hence repressing *CDH1* expression.

We next confirmed the involvement of TGFB1 in leptin-induced EMT and CSC changes by co-treating cells with neutralizing antibody against TGFB1 (Ab-TGFB1). The induction of EMT by leptin as indicated by decreased *CDH1* mRNA expression could be mimicked by TGFB1 treatment, and was almost completely blocked by co-treatment of the cells with Ab-TGFB1 in both MCF7 and MCF10AT1 cells (Fig 7A and 7B). At the functional level, a similar effect of Ab-TGFB1 was also observed for the increased rate of cell migration after leptin treatment (Fig 7C and 7D) and for aldefluor activity in both cell lines (Fig 7E and 7F). These results clearly indicate a role for the TGFB1 receptor in the leptin-mediated changes in breast cancer cell characteristics. Fig 8 shows a schematic representing the proposed autocrine/paracrine TGFB1 signalling in breast cancer cells resulting from leptin exposure.

## Discussion

The association between obesity and poorer outcomes from breast cancer is clear, however the mechanisms underlying this are complex. In this study, four steps in the cancer progression process were found to be significantly altered by leptin, namely cell proliferation, EMT, invasiveness and cell migration. Leptin treatment in both MCF7 and MCF10AT1 cells increased proliferation (Fig 1), and altered *CDH1* and *SNAI2* expression indicating EMT (Fig 3). An increase in metastatic potential was confirmed by measuring decreased cell aggregation, increased cell migration, and increased invasiveness through matrigel (Fig 2). We also found leptin to increase the stemness of both cancer cell lines, as indicated by aldefluor activity, CD44/CD24 expression, and mammosphere formation (Fig 4). This suggests that higher circulating levels of leptin present in obese breast cancer patients might be contributing to the development of higher grade and more metastatic tumors by these direct actions of leptin on breast cancer epithelial cells. Interestingly, one study has reported higher leptin levels in breast cancer tissue compared to normal breast tissue from the same patient [35], suggesting elevated local production of leptin in the tumor microenvironment. This may arise from cancer associated fibroblasts (CAFs) which have been recently shown to secrete leptin, mediating cross-talk between CAFs and breast cancer cells to drive growth and invasion [36], mammosphere formation and stemness [37]. This extends the relevance of the adverse actions of leptin to non-obese women with normal circulating leptin levels.

Our findings are consistent with those of Yan et al. who reported that leptin induces EMT in several breast cancer cell lines treated in serum-starved conditions for only 4 days [28]. Conditions of leptin treatment in published studies varies considerably, from acute (1–4 days) physiological levels (50–200 ng/ml), to supraphysiological concentrations (up to 1600 ng/ml) for 2 days [38], often in serum starvation conditions. Our conditions are low serum for 14 days, a chronic treatment protocol that is perhaps more representative of the actual clinical condition of obesity, and may explain why the magnitude of many of the changes we observed are less than those reported by Yan et al. Some epidemiological studies have suggested that there are substantial differences between the association of obesity and breast cancer based on premenopausal and postmenopausal status [39–41] and have linked obesity as a risk factor in postmenopausal women via the production of estrogens by adipose tissue. Further, there is cross-talk between estrogen and leptin signalling [21], such that leptin may increase estrogen levels by increasing aromatase expression [42], and estrogen may enhance the actions of leptin via increasing leptin-induced STAT3 activity [43]. However, our study and that of Yan et al. indicate clearly that leptin can directly induce adverse changes in breast cancer cell characteristics, and these effects are not restricted to estrogen-dependent breast cancer cells, as they occurred in both hormone receptor positive (MCF7) and negative (MCF10AT1, MDA-MB-468 and MDA-MB-231) cell lines. This is also supported by Zheng et al. who describe a mesenchymal-to-epithelial transition, reduced tumor spheres and tumour outgrowths in MDA-MB-231 cells after LEPR silencing [44]. These studies were performed in serum-free media, further supporting the EMT actions of leptin to be independent of steroid receptor signalling, although a contribution from phenol red in the media cannot be excluded. Further studies using charcoal stripped serum or ER knockdown strategies are warranted to fully elucidate the contribution of ER to the EMT actions of leptin mediated by TGFB or other signalling pathways, as clinical ER pathway antagonists may be useful in combination therapy strategies.

The above studies confirm that chronic leptin exposure can produce the same EMT- and CSC-like changes reported by others who used acute leptin treatment. We have also identified binding of TGFB1 to its receptor as a new pathway contributing to the leptin-induced EMT and CSC effects on breast cancer cells. We demonstrated that leptin treatment of breast cancer cells can increase TGFB1 expression (Fig 6). Further, when TGFB1 is blocked by use of a TGFB1 neutralizing antibody, the actions of leptin promoting EMT and CSC phenotypes are also blocked, thus strongly suggesting that TGFB1 is involved in mediating the leptin-driven EMT/CSC phenotype (Fig 7). While our mechanistic experiments on TGFB signalling are not extensive, the fact that we can show a significant contribution to the EMT actions of leptin even in the context of low serum conditions and some background of estrogen receptor activity points to the likely significance of our finding in a physiological setting, and warrants further investigation in future studies. We propose that this occurs in an autocrine manner, where leptin induces TGFB1 expression and secretion, which then acts on TGFBRI and TGFBRII receptors of breast cancer cells (Fig 8). While the role of TGFB1 in EMT is well established, its placement downstream of leptin signalling is evident in only limited studies. A diverse range of normal cells show increased secretion and/or mRNA levels of TGFB1 after acute in vitro treatment with leptin [45–47]. In prostate cancer cell lines, leptin treatment increased TGFB1 secretion 2-3-fold in a time and dose dependent manner [48], and in A549 lung cancer cells, leptin exposure increased TGFB1 expression, enhanced metastasis and EMT induction that was inhibited by siRNA against TGFB1 [49]. Note our chronic leptin treatment regime, unlike many acute treatment studies, would allow adequate time for TGFB1 to be transcribed, translated, secreted and activated, allowing observation of the maximal contribution of TGFB to EMT [29]. Thus, with both expression and intervention studies in two breast cancer cell lines, we extend the significance of the TGFB1 pathway to being a downstream mediator of leptin signalling for EMT / CSC changes in breast cancer cells.

Cross talk between TGFB1 and leptin signalling pathways may amplify the effects of either of these ligands and call for combination therapies. In addition to TGFB1 being downstream of leptin, interactions between TGFB1 and leptin signalling have been observed in kidney fibroblasts where leptin treatment strongly enhanced Smad2/3 phosphorylation by TGFB1, indicating leptin can be a co-factor for TGFB1 signalling in vitro [50]. JAK/STAT3 is a major signalling pathway for leptin action [51] and JAK/STAT3 can be a signalling intermediate for EMT via TGFB1 in cancer [52]. Leptin-induced EMT can occur through β-catenin activation via Akt/GSK3 and MTA1/Wnt1 protein-dependent pathways [28]. Zhou et al. demonstrated that TGFB1 activates β-catenin-dependent signalling and synergizes with the Wnt/β-catenin signalling pathway to induce EMT [53]. CAFs could also respond to leptin to increase TGFB1 for paracrine signalling [21] thus targeting TGFB1 and leptin signalling could target both cancer cell and CAF contributions to these synergistic metastatic pathways in tumors.

The relationship between TGFB1 and leptin signalling leads to the suggestion that treatment of obese breast cancer patients with drugs that can reduce leptin levels or antagonize TGFB1 signalling may be beneficial in prevention of metastatic disease. Several peptide antagonists of LEPR (allo-aca, PEG-LPrA2 and LDFI) have been shown independently to be effective against both estrogen receptor positive (MCF7) or negative (MDA-MB-231) breast cancer xenograft models [54–56]. Clinical development of TGFB1 inhibitors is more advanced, with several agents in phase III clinical trials (reviewed in [57]). These TGFB1 inhibitors can be grouped in 3 mechanistic categories–anti-TGFB antibodies, anti-sense oligonucleotides against TGFB, and TGFB receptor kinase inhibitors [57]. Our studies support consideration of two of these categories for use to reduce the impact of leptin on cancer progression. We have demonstrated that antagonizing the TGFB-TGFB-receptor interaction can reduce the EMT-promoting effects of leptin (Fig 7), an effect that should be able to be mimicked with the clinical anti-TGFB antibodies. Further, we have demonstrated that leptin treatment increased intracellular TGFB protein (Fig 6), which should be able to be antagonized with TGFB anti-sense oligonucleotides. We have not investigated (eg. via showing increased p-SMAD2/SMAD3) the activation of the TGFB receptor kinase by leptin and so cannot comment on the rationale for applying the kinase inhibitors against the actions of leptin. Future studies demonstrating the reduction of the actions of leptin with these clinical TGFB-targeting agents would be very valuable. More immediately though, the TGFB inhibitor trials do not specifically target obese patients, but our findings suggest that stratification of patients according to body mass index (BMI) would be of interest, as obese patients may derive more benefit. However, it must be cautioned that TGFB has both proliferative and growth inhibitory actions, depending on the concentration and cell context (normal vs cancerous), thus while long term use of antagonists may reduce metastatic disease developing from existing cancers, it may also come with increased risk of developing new tumors [58]. The anti-diabetic drug metformin is another therapeutic option that can decrease leptin levels in normal weight or obese non-diabetic people [59–62], inhibit TGFB signalling [63, 64], inhibit STAT3 signalling [65] and block EMT/CSC induction [66]. Thus, metformin may work through several pathways identified in this report to reduce metastases and improve outcomes in obese and non-obese patients.

This study strengthens the potential significance of leptin in breast cancer progression and identifies the TGFB1 receptor as a functionally important and novel route mediating the actions of leptin in promoting metastasis. This opens up new therapeutic opportunities via agents that block TGFB1 receptor and reduce TGFB1 expression that are in currently in phase III clinical trials in other malignancies. A better understanding of the pathways involved in poor cancer outcomes due to obesity will allow us to use rational combinations of agents such as TGFB1 antagonists, anti-estrogens, and metformin for improving outcomes in obese breast cancer patients.
